# Stem Cells from Dental Pulp: What Epigenetics Can Do with Your Tooth

**DOI:** 10.3389/fphys.2017.00999

**Published:** 2017-12-06

**Authors:** Beatriz A. Rodas-Junco, Michel Canul-Chan, Rafael A. Rojas-Herrera, Clelia De-la-Peña, Geovanny I. Nic-Can

**Affiliations:** ^1^CONACYT-Facultad de Ingeniería Química, Campus de Ciencias Exactas e Ingeniería, Universidad Autónoma de Yucatán, Mérida, Mexico; ^2^Facultad de Ingeniería Química, Campus de Ciencias Exactas e Ingeniería, Universidad Autónoma de Yucatán, Mérida, Mexico; ^3^Unidad de Biotecnología, Centro de Investigación Científica de Yucatán, Mérida, Mexico

**Keywords:** cell differentiation, dental pulp, DNA methylation, stem cells, histone modification, miRNAs, multilineage potential

## Abstract

Adult stem cells have attracted scientific attention because they are able to self-renew and differentiate into several specialized cell types. In this context, human dental tissue-derived mesenchymal stem cells (hDT-MSCs) have emerged as a possible solution for repairing or regenerating damaged tissues. These cells can be isolated from primary teeth that are naturally replaced, third molars, or other dental tissues and exhibit self-renewal, a high proliferative rate and a great multilineage potential. However, the cellular and molecular mechanisms that determine lineage specification are still largely unknown. It is known that a change in cell fate requires the deletion of existing transcriptional programs, followed by the establishment of a new developmental program to give rise to a new cell lineage. Increasing evidence indicates that chromatin structure conformation can influence cell fate. In this way, reversible chemical modifications at the DNA or histone level, and combinations thereof can activate or inactivate cell-type-specific gene sequences, giving rise to an alternative cell fates. On the other hand, miRNAs are starting to emerge as a possible player in establishing particular somatic lineages. In this review, we discuss two new and promising research fields in medicine and biology, epigenetics and stem cells, by summarizing the properties of hDT-MSCs and highlighting the recent findings on epigenetic contributions to the regulation of cellular differentiation.

## Introduction

Human life begins with a single totipotent cell (zygote) that divides into two equal cells, then four, then eight, and so on until the morula arises. Cell divisions continue millions of times to generate an embryo. Cells in the division process increase their cellular mass and expand their phenotypic diversification (Moris et al., 2016). Cell specialization and differentiation potential gradually increase throughout development. During this process, specific adult tissues, such as the bone marrow, skeletal muscle, and fat, contain stem cells that must decide between self-renewal or gaining a new cell identity. Thus, adult stem cells can maintain tissue homeostasis through self-renewal or can repair injuries or replace damaged cells through cell differentiation (Rumman et al., 2015). The ability to replace damaged cells in an organism has caught the interest of scientists. Currently, researchers are particularly interested in understanding the regenerative power of these cell populations and their potential use for immunotherapy or the regenerative treatment of various human disorders. However, whether all organs contain a pool of stem cells to sustain tissue turnover has not been determined. In recent years, several populations of adult stem cells have been identified in diverse tissues, including the skin, cartilage, intestine, blood, dental pulp, and mammary epithelial cells, among others (Gronthos et al., 2000; Bianco, 2014; Deng et al., 2015). All of these tissues are good reservoirs of stem cells, but the mechanisms related to cellular reprogramming remains to be discovered.

Recent studies suggest that the homeostasis between the self-renewal of adult stem cells and their transition into more specialized cells is regulated by epigenetic modifications (Deng et al., 2015; Rinaldi and Benitah, 2015). Such epigenetic modifications are orchestrated by posttranslational histone modifications, DNA methylation and noncoding RNAs, which converge to modulate chromatin structure, allowing or preventing the access of transcriptional machinery to genomic information. Therefore, these mechanisms guarantee that genetically identical cells display a great variety of phenotypes with defined functions, whereas the dysfunction of specific epigenetic regulators can lead to the development of diseases, such as cancer (Van der Meulen et al., 2014; Zhang T. Y. et al., 2015; Avgustinova and Benitah, 2016).

**Figure 1:**
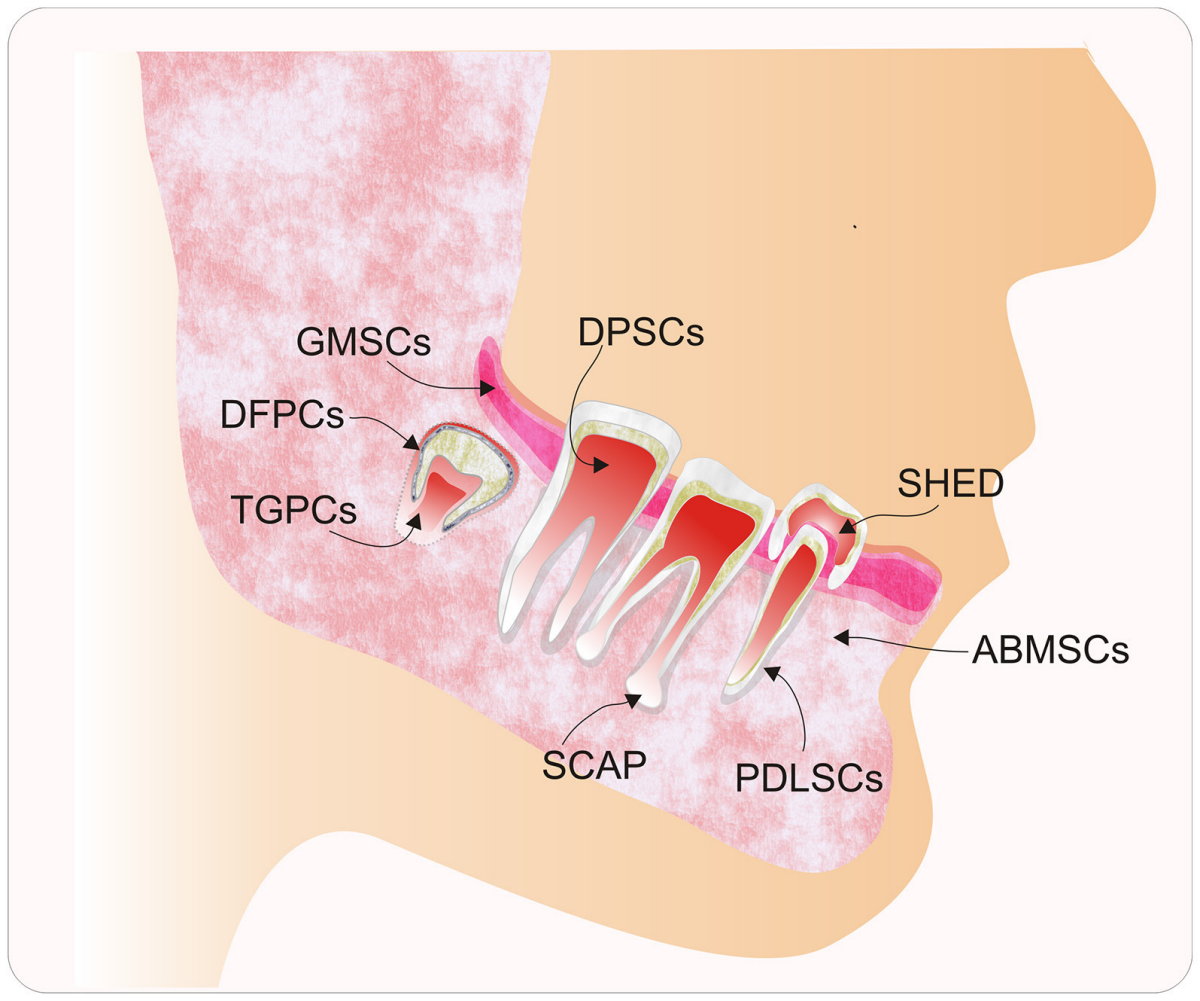
Diagram of the main sources of human dental tissue-derived mesenchymal stem cells (hDT-MSCs). Typical populations of stem cells that have been isolated and characterized from the oral cavity, including alveolar bone marrow-derived mesenchymal stem cells (ABMSCs), dental follicle progenitor cells (DFPCs), dental pulp stem cells (DPSCs), gingival-derived mesenchymal stem cells (GMSCs), periodontal ligament stem cells (PDLSCs), stem cells from the apical papilla (SCAP), stem cells from human exfoliated deciduous teeth (SHED), and tooth germ progenitor cells (TGPCs).

**Figure 2:**
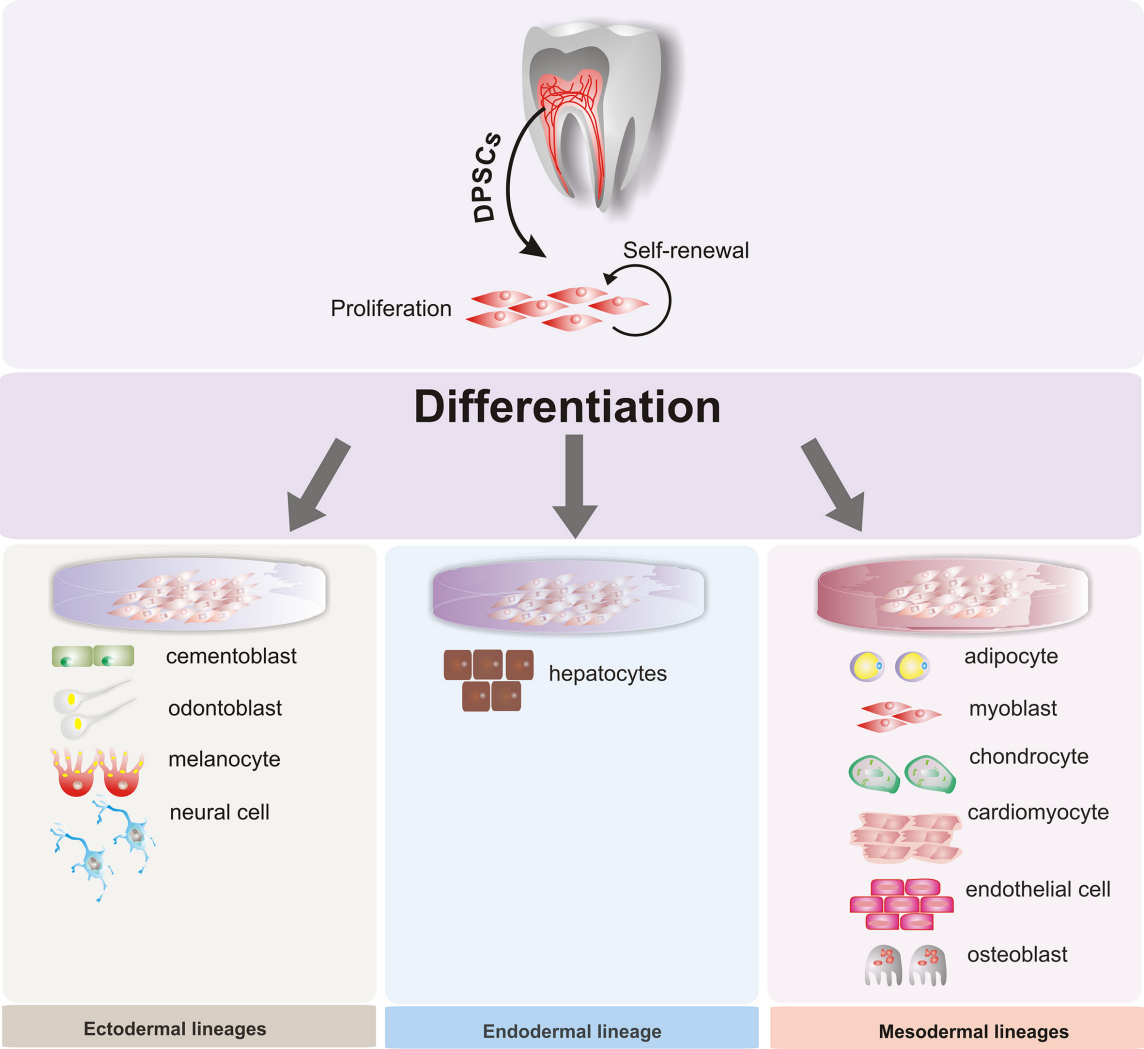
Multilineage potential of human dental pulp stem cells. Dental pulp stem cells (DPSCs) have the capacity to differentiate under appropriate conditions into different somatic cell types of the three germ layers: endoderm, mesoderm, and ectoderm.

In particular, human dental tissues, including the dental pulp, dental follicle, periodontal ligament, bone marrow, and others (Figure 1), have been identified as a promising source of mesenchymal stem cells (MSCs). Under suitable conditions, they can differentiate into multiple cell types, such as neural cells, osteocytes, and chondrocytes, among others (Figure 2). These MSCs could be used for therapeutic and regenerative treatments (Liu et al., 2015; Yang et al., 2017). This important ability represents a novel way to repair or regenerate damaged tissues, and this promising field of study could help to resolve some unresolved questions about cell fate reprogramming through epigenetics. In this review, we summarize recent progress on human dental tissue-derived MSCs (hDT-MSCs), focusing on their high plasticity for development into distinct cell lineages, as modulated by epigenetic mechanisms.

## From tooth to dental tissue-derived stem cells

Although embryos do not have visible teeth, human tooth development begins at the fifth week of gestation (Koussoulakou et al., 2009). Tooth development begins in the primary epithelial band, where the dental lamina is formed. Thereafter, a series of morphological changes generated by cellular proliferation in the dental lamina allows the formation of the bud stage. This bud is formed through an invagination of oral epithelial cells into the ectomesenchymal zone, generating an ovoidal-like structure, which stimulates mesenchymal cell condensation around the bud (reviewed by Volponi et al., 2010; Fawzy El-Sayed et al., 2013; Yildirim, 2013).

The bud stage continues its development through epithelial bud cell proliferation, and differentiation allows formation of the cap stage. In the cap stage, crown histodifferentiation is initiated from the enamel knot, an epithelial cell that acts as a signal center and plays an organizing role, which enables the generation of both the dental cap and the dental papilla (derived from condensed ectomesenchymal cells), from which dental pulp originates. At the bell stage, the terminal differentiation of the odontoblast and ameloblasts occurs, producing dentine and coordinating enamel deposition, respectively. Reciprocal signals between the odontoblast and ameloblasts are crucial to supporting tooth development, whereas the coordination of bone resorption and root development are important for tooth eruption (Volponi et al., 2010; da Cunha et al., 2013).

Primary teeth are completed in 5- to 6-year-old children, and these teeth are lost around age 12, whereas permanent teeth begin to erupt at age 6 and can continue erupting until age 17–25 (Volponi et al., 2010; Mushegyan et al., 2014). Inside each primary or permanent tooth is dental pulp (DP), which is an innervated, highly vascularized soft tissue that provides vitality to the tooth. This tissue is surrounded by dentine, the tooth crown (a zone exposed to oral cavity) and cementum (a connective tissue that surrounds the tooth root). The main functions of DP include the generation of dentine and the maintenance of its biological and physiological vitality in response to traumatic injuries, physical stimulus, or bacterial infections (Ravindran and George, 2015; Tatullo et al., 2015). The ability of DP cells (DPCs) to form reparative dentin resides in the regenerative potential of their undifferentiated mesenchymal cells, which can migrate toward the wound site and redifferentiate into new odontoblasts (Yamamura, 1985). This ability suggests that DP contains odontogenic progenitor cells or stem cells that are involved in the regeneration process. In 2000, it was shown for the first time that human DP cells from the third molar exhibited high clonogenic capacity as well as great multilineage potential (Gronthos et al., 2000). Due to their properties of such cells, these were called dental pulp stem cells (DPSCs). Currently, several stem cell populations have been isolated and characterized from different parts of the oral cavity, including alveolar bone marrow-derived mesenchymal stem cells (ABMSCs), stem cells from human exfoliated deciduous teeth (SHED), stem cells from the apical papilla (SCAP), periodontal ligament stem cells (PDLSCs), gingival-derived mesenchymal stem cells (GMSCs), tooth germ progenitor cells (TGPCs), and dental follicle progenitor cells (DFPCs), which are involved in the development of both primary and permanent teeth. These cells express several mesenchymal markers, such as CD29, CD73, CD90, and CD105 among others, as well as embryonic markers, including *SOX2, NANOG*, and *OCT4*, and they differentiate into multiple cell lineages (Table 1). In addition, some dental stem cells have shown more embryogenic-like characteristics than bone marrow stem cells, umbilical cord, or others (Miura et al., 2003; Hilkens et al., 2013; Ren et al., 2016). Therefore, MSCs derived from the oral cavity represent an invaluable resource for the development of future clinical-grade cells.

**Table 1:** Summary of characteristics of human dental tissue-derived mesenchymal stem cells.

**Human dental tissue-derived mesenchymal stem cells**								
**Marker**	**DPSCs**	**SHEDS**	**SCAP**	**PDLSCs**	**GSCs**	**DFPCs**	**TGPC**	**ABMSCs**
CD9	+			+		+		
CD10	+	+		+		+		
CD13	+	+	+	+		+		
CD29	+	+	+	+	+	+	+	
CD44	+	+	+	+	+	+	+	
CD56	+		+					
CD59	+			+		+		
CD71	+							+
CD73	+	+	+	+	+	+	+	+
CD90	+	+	+	+	+	+	+	+
CD105	+	+	+	+	+	+	+	+
CD106	+	-	+	+	+	+	+	
CD117	+	+	–		–			
CD146	+	+	+	+	+			
CD166	+	+	+	+	+	+	+	
CD271	+					+		
CD11b	–	–						–
CD14	–	–	–	–			–	–
CD19	–			–				–
CD31	–	–		–		-		
CD34	–	–	-	–	–	–	–	–
CD43	–	–						
CD45	–	–	-	–	–	–	–	–
CD150			-					
OCT3/4	+	+	+	+	+	+	+	
SOX2	+	+	+	+	+	+	+	
NANOG	+	+		+	+	+	+	
c-Myc	+	+	+				+	
KLF4	+		+				+	
LIN28	+							
STRO-1	+	+	+	+	+	+	+	+
SSEA-3	+	+						
SSEA-4	+	+	+	+	+			
TRA-1-60	+	+						
Dif. Potential	Adipo, neuro, odonto, osteo, myo, endo, chondro, cardio, melano, hepato.	Adipo, neuro, odonto, osteo, myo, endo, chondro.	Adipo, neuro, odonto, osteo, chondro, hepato.	adipo, odonto, osteo, chondro, cemento.	Adipo, neuro, osteo, chondro.	Adipo, neuro, osteo, chondro, cemento, hepato.	Adipo, neuro, odonto, osteo, chondro, endo, hepato.	Adipo, osteo, chondro.
Refs.	Gronthos et al., 2000; Kadar et al., 2009; Karaoz et al., 2010; Atari et al., 2011, 2012; Ferro et al., 2012b; Ishkitiev et al., 2012	Miura et al., 2003; Kerkis et al., 2006; Wang et al., 2010, 2012; Akpinar et al., 2014; Trivanovic et al., 2015	Sonoyama et al., 2006, 2008; Huang et al., 2009; Yam et al., 2015	Seo et al., 2004; Tarle et al., 2011; Navabazam et al., 2013; Rodriguez-Lozano et al., 2013; Lei et al., 2014; Liu et al., 2016	Lindroos et al., 2008; Zhang et al., 2009; Marynka-Kalmani et al., 2010; Tang et al., 2011; Wang et al., 2011	Kemoun et al., 2007; Lindroos et al., 2008; Huang et al., 2009; Gopinathan et al., 2013; Navabazam et al., 2013	Huang et al., 2008; Ikeda et al., 2008; Yalvac et al., 2010a,b; Yalvac et al., 2013	Matsubara et al., 2005; Park et al., 2012; Pekovits et al., 2013; Mason et al., 2014

*+, Positive marker expression; –, Very weak or lack of marker expression; adipo, adipocytes; cardio, cardiomyocytes; chondro, chondrocytes; endo, endothelial cells; hepato, hepatocytes-like cells; melano, melanocytes; myo, myoblast; neuro, neuronal cells; odonto, odontoblast; and osteo, osteoblast. ABMSCs, Alveolar bone marrow-derived mesenchymal stem cells; DFPCs, dental follicle progenitor cells; DPSCs, dental pulp stem cells; GMSCs, gingival-derived mesenchymal stem cells; PDLSCs, periodontal ligament stem cells; SCAP, stem cells from the apical papilla; SHED, stem cells from human exfoliated deciduous teeth; and TGPCs, tooth germ progenitor cells*.

## Isolation of dental tissue-derived mesenchymal stem cells

Recently, the oral cavity has received increased attention due to the minimally invasive harvesting of its somatic stem cells, which are present in several dental tissues, including the dental pulp, dental follicle, periodontal ligament, alveolar bone marrow, gingiva, apical papilla, and tooth germ (Figure 1). Dental stem cells are generally obtained by two methods: outgrowth from explants and the enzymatic digestion of dental tissues (Karamzadeh et al., 2012). While the explant method is based on the migration of stem cells from small tissue fragments and their ability to adhere to a plastic surface, the enzymatic digestion method involves the application of collagenase and dispase to dissociate cells from dental tissues and acquire single cell suspensions (Karamzadeh et al., 2012; Hilkens et al., 2013). Both methods have been used successfully to obtain different kinds of hDT-MSCs, including DPSCs, SCAP, SHED, and PLSCs (Martens et al., 2012; Zhang et al., 2012; Hilkens et al., 2013). Although the cells obtained by both methods are generally cultured in alpha minimum essential medium (α-MEM) supplemented with concentrations of fetal bovine serum (FBS) between 10 and 20% (Gronthos et al., 2000; Karamzadeh et al., 2012), a standardized protocol to isolate and individually culture each population of hDT-MSCs is lacking. It has been reported that variations among different culture media, such as MEM/F12, MEM-Low Glucose, and α-MEM, can influence the differential expression of stemness markers, such as *CD105, CD73*, and *OCT3/4* (Lizier et al., 2012). Moreover, there are several issues with using FBS since it is commonly used to expand and induce differentiation from DPSCs into different lineages. Although FBS provides nutrients, vitamins, growth and attachment factors, hormones, and proteins, these factors can all vary among different lots of FBS. In addition, the possibility exist that viruses, prions, endotoxins, and mycoplasma, among others pathogens, could be present in the FBS and damage the valuable odontogenic stem cells; such pathogens may also represent a potential risk for disease transmission and xenogeneic immune responses (Pisciotta et al., 2012; Spina et al., 2016). To decrease or replace the use of FBS, other alternatives, such as autologous human serum (HS) and human platelet lysate (HPL), have been applied to maintain the stability and differentiation potential of MSCs (Bieback et al., 2009; Ferro et al., 2012a; Pisciotta et al., 2012; Marrazzo et al., 2016). Another recent option for the culture of dental cells is the use of New Zealand FBS (NZ-FBS), which is a clinical-grade serum approved for good manufacturing practices (GMP). The results have shown a significant improvement in cell growth and osteogenic differentiation potential as well as an increase in the expression of angiogenic factors on DPSCs (Spina et al., 2016). These improvements suggest that NZ-FBS might be a viable alternative to the FBS traditionally used in MSCs cultures. On the other hand, the use of HS improves the cell growth of DPSCs and provides a consistency in the expression of stem cell markers, as well as an osteoblastic potential similar to that provided by common differentiation protocols that use 10% FBS (Ferro et al., 2012a). Currently, the use of HS with GMP procedures has successfully promoted the proliferation and differentiation of DPSCs into osteoblasts and the generation of well-vascularized woven bone for the first time without the use of scaffolds (Paino et al., 2017). The application of this approach using GMP-approved HS might substantially improve the bone regeneration therapy, since the scaffolds often compromise the success of grafting. Furthermore, the use of HS has also been used recently to evaluate the potential of DPSCs for dental pulp tissue regeneration. DPSCs were found to expand in human serum and also to be able to regenerate *in vivo* DP without compromising the angiogenic and differentiation properties of DPSCs (Piva et al., 2017).

Recent studies have also reported that HPL supports the expansion of MSCs better than FBS does due to its enrichment in growth factors and cytokines (Marrazzo et al., 2016; Fernandez-Rebollo et al., 2017). At a low concentration of HPL (1%), DPSCs exhibited a good viability and proliferation profile. In addition, the osteogenic and chondrogenic differentiation capacity was also sustained at the same low concentration of HPL (Marrazzo et al., 2016). All these findings suggest that animal serum and exogenous growth factors could be avoided and replaced by either HS or HPL since the growth and the differentiation of DPSCs can be sustained. However, basic research studies need to be performed, which could contribute to a better understanding of human diseases as well as continually evaluating the therapeutic potential of hDT-MSCs for new applications in the fields of regenerative medicine or cellular therapy.

## Multipotent differentiation of human dental tissue-derived mesenchymal stem cells

In the early stages of embryogenesis, a group of cells known as neural crest cells (NCCs) arises from the ectoderm at the margin of the neural tube (La Noce et al., 2014a). These cells are a multipotent SC population, which is surpassed only by the inner cell mass cells of the blastocyst in their ability to contribute to cell types characteristic of all three germ layers (Kerosuo and Bronner, 2016). On the other hand, following extensive migration, the NCCs give rise to many different cell types within the embryo and therefore contribute to several systems such as the skin, heart, skull, cartilage, nerves, and teeth, among others (Xiao and Tsutsui, 2013; La Noce et al., 2014a). Furthermore, during tooth development, NCCs play a crucial role in the generation of the dental mesenchyme, which gives rise to most of the dental cells including dentin, dental pulp, periodontal ligament, alveolar bone, and others (Xiao and Tsutsui, 2013; Nuti et al., 2016). The fact that human dental stem cells have the ability to differentiate into multiple cell lineages, such as adipocytes, chondrocytes, osteoblasts, cardiomyocytes, melanocytes, neuronal cells, hepatocytes, and endothelial cells, among others (Table 1), strongly suggest that the different niches of SCs found in the oral cavity still retain certain embryonic attributes conferred through NCCs (Pisciotta et al., 2015a; Nuti et al., 2016). Therefore, hDT-MSCs have the potential to provide new therapeutic strategies that can be used for basic developmental research, to serve as a model to more deeply understand pediatric diseases and disorders and to enable applications in drug screening, tissue engineering, and cell therapy (La Noce et al., 2014b; Aurrekoetxea et al., 2015; Ducret et al., 2015). However, it must be considered that stem cell quantity can be affected by odontogenesis as well as by the heterogeneity of human dental stem cells, since different subsets of stem cells may be distinguished based on the expression of distinct surface markers, some of which enable the isolation of stem cells populations with different behavior in terms of proliferation and differentiation (Hara et al., 2013; Pisciotta et al., 2015a).

Briefly, DPSCs express several cell surface antigens, such as CD29, CD73, CD90, and CD105, as well as different embryonic markers, including *OCT4, NANOG*, and *KLF4* among others. Moreover, DPSCs possess immunomodulatory properties, a good proliferative rate and high potential to differentiate into more than eight cellular types (Table 1) (Gronthos et al., 2000; Karaoz et al., 2011). Therefore, DPSCs have been considered a good option for applications in clinical trials, regenerative medicine, and tissue engineering (Paino et al., 2010; Woloszyk et al., 2016).

SHED are mesenchymal cells derived from deciduous teeth that are naturally replaced to give rise to permanent teeth. These stem cells exhibit a high proliferative rate and clonogenicity; although they can differentiate into adipocytes, neural cells, and odontoblasts, it appears that SHEDs, unlike DPSCs, cannot differentiate into a dentin-pulp like complex (Miura et al., 2003; Atari et al., 2012). However, several pluripotent stem markers, such as *OCT4, SOX2*, and *NANOG*, as well as different growth factors, including fibroblastic growth factor, transforming growth, and connective growth factor, which promote stemness and cell proliferation, are upregulated in SHED (Nakamura et al., 2009). Taken together, this evidence suggests that the main difference between deciduous and permanent teeth might be in the immature development stage of deciduous teeth. In this sense, it has been reported that a population of MSCs (also isolated from deciduous teeth) have similar characteristics to human ES cells (Kerkis et al., 2006). These MSCs, designated immature DPSCs (IDPSCs), display several pluripotency markers (*OCT4, SSEA-3, SSEA-4, TRA-1-60, TRA-1-80*, and *NANOG*), and they are easily induced to differentiate into neuronal cells, chondrocytes, osteocytes, and myocytes (Kerkis et al., 2006; Lizier et al., 2012), suggesting that multiple niches of SCs might be present in DP tissue, which could contribute to different levels of cell plasticity over time (Lizier et al., 2012).

The root apex of developing teeth also contains a unique population of stem cells designated SCAP (Sonoyama et al., 2008). These cells are located in the apical papilla, a cell-rich zone that contributes to tooth formation and pulp tissue (Figure 1). SCAP express typical MSC markers and exhibit higher proliferation and better mineralization but lower adipogenic differentiation than DPSCs; however, they can differentiate into odontoblasts, osteoblasts, neural cells, and hepatocyte-like cells (Sonoyama et al., 2008; Patil et al., 2014; Zhang et al., 2016).

Ikeda et al. (2008) used wisdom teeth at the late bell stage to isolate and identify novel dental stem cells called TGPCs. These stem cells have spindle-shaped cell morphology with reduced cytoplasm. Moreover, TGPCs have a mesenchymal phenotype that can differentiate into three germ layers and are thought to have novel primitive stem cell properties, as they were able to express pluripotency-associated genes, such as *NANOG, OCT4, SOX2, KLF4*, and c-*MYC* (Ikeda et al., 2008; Yalvac et al., 2010a).

The dental follicle is a mesenchymal tissue that surrounds the tooth germ (Figure 1), and it is thought to contain SCs from which cementoblasts, periodontal ligament cells, and alveolar bone can be derived. These stem cells, also known as DFSCs, have been isolated from impacted third molars. Like DPSCs, DFSCs exhibit high telomerase activity and are capable of differentiating into osteoblasts, chondrocytes, and adipocytes, among others (Table 1). The presence of SCs has been shown in the dental follicle (Huang et al., 2009; Honda et al., 2010).

As its development continues, the dental follicle gives rise to the periodontium, a soft, complex connective tissue from which another stem cell population, designated PDLSCs, can be isolated. PDLSCs are heterogeneous cells that participate in maintaining periodontium homeostasis, whereas *in vitro*, they have the ability to differentiate into chondrocytes, adipocytes, osteoblast, neural cells, and hepatocytes (Seo et al., 2004; Bartold et al., 2006). Additionally, it was found that CD146^+^ cells isolated from PDLSCs display better clonogenicity, multipotency, and regenerative potential than heterogeneous PDLSCs, suggesting that a specific population of PDLSCs might help in effectively combating periodontitis (Seo et al., 2004; Cho et al., 2016).

Unlike DPSCs, TGPSCs, DFPCs, or the SCAP, which can only be obtained as a supply of stem cells by tooth or pulp extraction, gingival tissue and alveolar bone represent alternative sources of stem cells with great potential for future maxillofacial applications, as these cells can be obtained from biopsies without compromising the loss of a valuable tooth (Fawzy El-Sayed et al., 2013). For instance, gingival tissue has remarkable regenerative and wound healing capacity; therefore, it has been considered a good source of stem cells called GMSCs (Irwin et al., 1994). *In vitro*, GMSCs exhibit self-renewal, clonogenicity and multipotent differentiation into adipocytes, chondrocytes, osteocytes, and neural cells (Rao S. R. et al., 2016; Van Pham et al., 2016). Mesenchymal progenitor cells that are derived from alveolar bone are referred to as ABMSCs (Figure 1); they exhibit a fibroblast-like, spindle-shaped morphology and show adherence to plastic, colony formation and high expression of CD73, CD90, and CD105. Like other dental MSCs, ABMSCs can also differentiate into adipocytes, osteoblasts and chondrocytes (Matsubara et al., 2005; Pekovits et al., 2013). In addition, their capacity to regenerate new bone *in vivo* (based on mouse transplant studies with ABMSCs) indicates that they could be used to improve bone defects.

On the other hand, it is clear that aging can affect stem cell function, since cell viability in terms of proliferation, migratory potential, and differentiation declines as age increases. This fact implies that specific culture conditions must be used to maintain stem cell activity (Zhang et al., 2012). For instance, even though DP contains a population of pluripotent-like stem cells (DPPSCs) with similar gene expression to induced pluripotent stem cells (iPSCs), the DPPSCs derived from 58-year-old donors must be cultured in the presence of leukemia inhibitor factor, epidermal growth factor, and platelet dermal growth factor to maintain the expression of embryogenic markers (Atari et al., 2011, 2012). Likewise, the use of hypoxia (3% of O_2_ and 5% CO_2_) in the DPC culture of samples from an older patient's teeth (age 62) was shown to effectively decrease the population doubling time and increase the proliferation rate and the expression of *STRO-1*, an early MSC marker. This observation suggests that such methods might be applied to recover and increase the low populations of SCs present in DP tissue independently of tooth age (Iida et al., 2010). Furthermore, Murakami et al. (2013) developed a method to isolate DPSCs with a higher proliferative rate and stability. The method is based on the application of granulocyte colony stimulating-factor (GCSF), which induces the mobilization of SCs to rescue and maintain different properties of DPSCs, including high proliferation rate and cellular stability, from aged donors (Murakami et al., 2013). Although these results appear encouraging, several obstacles must be addressed to enhance the efficiency of hDT-MSCs in clinical trials, including the standardization of isolation, enrichment, and cellular expansion techniques to obtain sufficient amounts of stem cells without compromising their stemness. In addition, more studies need to be conducted to gain insights into the molecular mechanisms that promote the multilineage capacity of hDT-MSCs to direct their differentiation into specific functional cells.

## Therapeutic applications of dental tissue-derived mesenchymal stem cells

The main application of dental stem cells has been the regeneration of oral and maxillofacial defects. For example, tissue engineering strategies to regenerate the dental pulp, periodontal complex, or tooth have begun to provide promising results, and these results have recently been reviewed elsewhere (Ravindran and George, 2015; Yang et al., 2017). However, the capability of dental stem cells to differentiate into multiple cell lineages (Figure 2) suggests that these mesenchymal cells have tremendous regenerative potential for repairing the brain, heart, liver, eye, bone, and muscle disorders (Ducret et al., 2015; Liu et al., 2015; Nuti et al., 2016). In this context, the mechanism of integration and the functions of dental stem cells are being extensively investigated in animal models before these cell populations can be employed in clinical therapy. For instance, the degeneration and loss of function of neurons in neurodegenerative diseases leads to progressive movement disorder (Parkinson's disease), memory loss (Alzheimer's disease), or loss of sensitivity and paralysis (spinal cord injury). Although effective treatments to restore the function of nerve cells do not yet exist, the capability of dental stem cells to differentiate into neural cells might represent a great advance in the field of neuroscience. For example, it was shown that the transplantation of dopaminergic neurons (DN) obtained through SHED-derived neural-like spheres in a rat model partially alleviated Parkinson's disease. The effects of DN in behavior tests showed that these cells were able to adequately express *TYROSINE HYDROXYLASE* (TH), a key element of dopamine synthesis. The grafting of DN into the striatum also resulted in the TH-positive cells and contributed to re-innervation of the host striatum in parkinsonian rats, suggesting that SHED could be an important resource for the treatment of neurodegenerative disease (Wang et al., 2010). DPSCs also produce and secrete various neurotrophic factors, including nerve growth factor, brain-derived neurotrophic factor, and glial cell line-derived neurotrophic factor, that can protect and promote the survival of DN against 6-hydroxy-dopamine, which is a neurotoxin for dopamine and norepinephrine neurons that causes neuronal death (Nosrat et al., 2004). In fact, neuroprotective effects of DPSC were also shown *in vitro* models with Alzheimer's disease (Apel et al., 2009). Furthermore, DPSCs can stimulate the long-term regeneration of nerves in damaged spinal cords (Sakai et al., 2012). This hypothesis was tested in completely severed spinal cords by transplanting DPSCs into rats. The results demonstrated that these stem cells promoted the regeneration of transected axons by directly inhibiting multiple axon growth inhibitors and by preventing the apoptosis of neurons, astrocytes, and oligodendrocytes. To evaluate whether DPSC-derived neuronal precursors can integrate into the central nervous system, labeled DPSCs were transplanted into the rats' cerebrospinal fluid. Interestingly, it was found that DPSCs migrated into various zones of the brain and exhibited neuronal features and voltage-dependent Na^+^ and K^+^ channels, suggesting that DPSCs may serve as a useful source of neurogenesis and gliogenesis *in vivo* (Kiraly et al., 2011). Likewise, the ability of STRO1^+^/c-kit^+^/CD34^+^ DPSCs to differentiate toward Schwann-cell like cells might provide an alternative therapeutic approach for the treatment of nerve injuries. For instance, it has been shown that this subpopulation of stem cells transplanted into a rat model with peripheral nerve injury, can be integrated into the sciatic nerve defects contributing with fibers regeneration by significantly increasing the number of myelinated axons (Carnevale et al., 2017). The results observed in these works could be indicated dental stem cells are a promising alternative for the peripheral nerve regeneration, as well as treatments of central nervous system disorders.

On other hand, although bone marrow MSCs have been widely used to study myocardial cell differentiation for cardiovascular regeneration for years (Singh et al., 2016), DPSCs have emerged as a new source of MSCs for the repair of myocardial infarction (MI) and other diseases. For instance, a study on the engraftment of green fluorescent protein (GFP)-DPSCs in rats with induced MI showed improved cardiac function after 4 weeks of treatment and concluded that DPSCs induce an increase in angiogenesis due to their ability to secrete multiple proangiogenic apoptotic factors, including vascular endothelial growth factor (VEGF) (Gandia et al., 2008). In addition, it has been shown that DPSCs have the ability to enhance vessel attraction and improve the neovascularization process, a crucial step for tissue repair and regeneration. Woloszyk et al. (2016) determined that silk fibroin scaffolds seeded with DPSCs attracted vessels from the chicken embryo chorioallantoic membrane, guaranteeing the homogeneous distribution of vessels in the scaffold. These results suggest that DPSCs possess interesting properties, including high angiogenic potential and the capacity to enhance the vascularization process, that must be further studied to create new strategies to improve tissue regeneration.

IDPSCs are particularly interesting because they have been used to improve the clinical condition of Duchenne muscular dystrophy (DMD). By the systemic delivery of IDPSCs into dogs with muscular dystrophy, Kerkis et al. (2008) showed that these dental stem cells can migrate and improve the myogenic potential of the host muscle, improving the clinical conditions of the dogs, which was likely due to the immunomodulatory effects of the IDPSCs. More recently, Pisciotta et al. (2015b) reported that the co-culture of DPSCs with a mouse myoblast cells lead to the formation of hybrid myotubes under myogenic differentiation conditions. The same authors also showed that the engraftment of resulting myogenic lineage improved the histopathology of DMD mice by promoting angiogenesis and by reducing fibrosis. This strongly suggests that DPSCs could be used for to improve and alleviate the muscle weakness of patients with DMD.

On the other hand, the therapeutic efficacy of IDPSCs has been evaluated in reconstruct of corneal damage. It is known that limbal stem cells (LSCs) are capable of reconstructing the corneal epithelium if injured; however, insufficient cell numbers for transplantation and rejection are the major obstacles to resolving this physical lesion. Fortunately, IDPSCs, which share some features with LSCs, can help solve this problem and may have important implications in designing new therapeutic applications. It has been reported that IDPSC transplantation into rabbits with induced partial or total LSC deficiency resulted in adequate formation of the stromal layer and stratified epithelium by contributing to the reconstruction of the eye surface. This result suggested that IDPSCs might be used as a potential alternative source of cells for corneal reconstruction (Monteiro et al., 2009; Gomes et al., 2010).

Finally, another type of dental stem cell that has positively impacted tissue regeneration is TGPCs. These cells can differentiate into hepatocyte-like cells; therefore, TGSCs might be considered an ideal source for understanding liver diseases or liver regeneration. For instance, Ikeda et al. (2008) showed that the engraftment of TGPCs yielded a significant therapeutic effect by preventing the progression of liver fibrosis and were able to restore liver function in tetrachloride-treated rats. This result suggested that TGPCs offer great possibilities for developing therapies for tissue repair and regeneration.

## Epigenetic modifications and their role in gene expression

**Figure 3:**
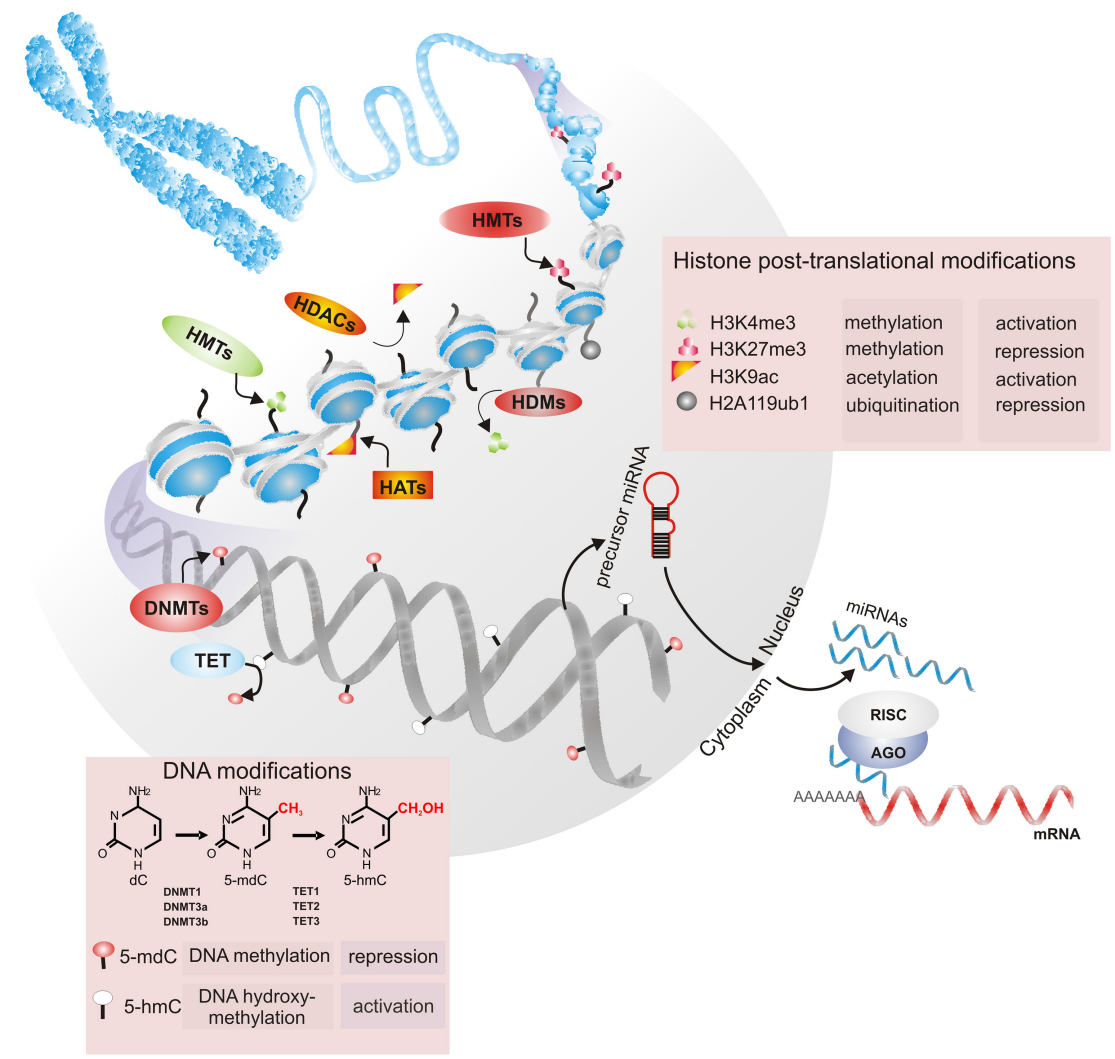
Chromatin's epigenetic markers are involved in its restructuration and remodeling. Dynamic changes in the conformational structure of chromatin are mediated by DNA methylation, histone posttranslational modifications (HPTMs) and noncoding RNAs. DNA methyltransferases (DNMT1, DNMT3a, and DNMT3b) catalyze the addition of a methyl group (-CH_3_) at position C5 of deoxycytosine (5-mdC). DNA methylation marks can be removed by members of the TET (TET1, TET2, and TET3) protein family through the conversion of 5-mdC to 5-hydroxymethyl-cytosine (5-hmC). Methylation, acetylation, and ubiquitination are HPTMs that may be dynamically regulated by histone methyltransferases (HMTs) and histone demethylases (HDMs) or histone acetyltransferases (HATs) and histone deacetylases (HDACs). During transcriptional repression, messenger RNA (mRNA) can be repressed by noncoding RNAs, such as miRNAs. Precursors of miRNAs are exported to the cytoplasm, where they are processed by Dicer; then, the miRNA is loaded into the RISC complex and binds to the mRNA target. Depending on the base pairing between the miRNA and the mRNA target, the binding can be destabilized, degraded or inhibited.

During embryogenic development, all lineages and cells that make up an organism are specified. Different organs and tissues are generated through two highly coordinated processes: increasing the number of cells and phenotypic diversification with high spatiotemporal precision (Moris et al., 2016). Considering that all cells in an organism have the same genetic information, the instructions to form a specific phenotype are driven by the cross-antagonistic expression of specific transcription factors (TFs) in response to environmental cues to promote cell fate changes or specify a cell lineage (Wu, 2014; Nashun et al., 2015). Such transcriptional events in stem cell renewal and fate decision events are regulated by epigenetic modifications, which change chromatin accessibility and dictate the proper instructions for cell identity (Table 2; Avgustinova and Benitah, 2016). Epigenetics can operate at different levels, such as by methylating cytosine residues in DNA structure, modifying posttranslational histone cores and interfering with transcriptional and translational information through noncoding RNAs (Figure 3; Brookes and Shi, 2014; Li et al., 2015; Zhang T. Y. et al., 2015).

**Table 2:** Epigenetic modifications and their role in *in vitro* differentiation of human dental tissue-derived stem cells.

**Cell population**	**Age of donors**	**Tooth type**	**Epigenetic mark**	**Chromatin modifiers**	**Biological response during cellular differentiation**	**References**
SHED	6–12	Primary teeth, third molars, and impacted third molars	5-mC	DNMTs	Decreased DNA methylation in promoter regions of *NANOG* and *OCT4* improve reprogramming of DPSC into iPS cells.	Oda et al., 2010; Yan et al., 2010
DPCs	15–18					
DPSCs	10–22					
SCAP	16–22					
PDLSCs	14–18	Premolars	5-mC	DNMTs	5-aza-dC decreases DNA methylation levels and rescue osteogenic differentiation capacity under high glucose conditions.	Liu et al., 2016
DPCs	18–25	Impacted third molar	5-mC	DNMTs	5-aza-dC decreases DNA methylation levels and suppresses cell proliferation, but improves odontogenic differentiation.	Zhang D. et al., 2015
DPCs	ND	Impacted third molar	5-hmC	TET	*TET1*-knockdown prevents cellular proliferation and odontogenic differentiation.	Rao L. J. et al., 2016
DPSCs	ND	ND	Ac	HDACs	*TSA* induces *HDAC3* downregulation and promotes proliferation and odontoblast differentiation.	Jin et al., 2013
DPSCs	ND	ND	Ac	HDACs	*HDAC2*-knockdown affects *OSTEOCALCIN* expression and impairs odontoblast differentiation.	Paino et al., 2014
DFCs, PDLCs	12–15	ND	Me	HMTs	DF cell progenitors contain high levels of H3K4me3. PDLCs are enriched with H3K9me3. DF cell differentiation into osteogenic cells leads to a switch from the H3K4me3 to the H3K9me3 mark.	Dangaria et al., 2011
DPCs	12–15	ND	Me	HMTs	In DF cells, odontoblast-related genes are enriched by H3K9me3 and H3K27me3, whereas in DPCs, the same loci are targeted by H3K4me3.	Gopinathan et al., 2013
DFCs						
SCAP	NE	Impacted third molar	Me	HDM	KDM6B is recruited at the promoter of *BMP2*, which leads to removal of the H3K27me3 mark to reactivate the function of BMP2 to regulate odontogenic differentiation.	Xu et al., 2013
SCAP	16–20	Impacted third molar	Me	HDM	*KDM2A* knockdown leads to increased H3K36me2 levels in the promoter of *SFR2* and enhances the osteo-/dentinogenic differentiation potential of SCAP.	Yu et al., 2016

*Ac, acetylation; DNMTs, DNA methyltransferases; DMTD, DNA methylcytosine dioxygenases; HDACs, histone deacetylases; HDM, histone demethylase; ND, not determined; Me, methylation; 5-aza-dC, 5-aza-2′-deoxycytidine; 5-mC, 5-methylcytosine; 5-hmC, 5-hydroxymethylcytosine; TET, ten-eleven translocation; TSA, trichostatin A. DFCs, Dental follicle cells; DPCs, dental pulp cells; DPSCs, dental pulp stem cells; PDLSCs, periodontal ligament stem cells; SCAP, stem cells from the apical papilla; and SHED, stem cells from human exfoliated deciduous teeth*.

Briefly, DNA methylation is a widely studied epigenetic tag correlated with changes in chromatin compaction, the transcriptional repression of promoters and the maintenance of cellular memory, among others (Baubec et al., 2015). Such modification is catalyzed by DNA methyltransferases (DNMTs), such as DNMT3a, DNMT3b, and DNMT1. It has been determined that both DNMT3a and DNMT3b carry out *de novo* methylation to mark unmethylated DNA, whereas DNMT1 maintains DNA methylation after replication (Li et al., 2007; Franchini et al., 2012). In addition to 5-methyl-2′-deoxycytosine (5-mdC), other DNA modifications include 5-hydroxymethyl-cytosine (5-hmC), 5-formylcytosine (5-fC), and 5-carboxylcytosine (5-caC). These 5-mdC oxidation derivatives are catalyzed by members of the TET (TET1, TET2, and TET3) protein family, which are suggested to be related to the maintenance of methylation-free regulatory regions in mammalian genomes (Plongthongkum et al., 2014). The appropriate balance between methylation and hydroxymethylation is thought to properly regulate gene expression.

At the histone level, amino acids in the N-terminal tails that extrude from the nucleosomal core can be post-translationally modified by methylation, acetylation, phosphorylation, ubiquitination, sumoylation, and other modifications. These modifications affect nucleosome stability and contribute to the relaxation or compaction of chromatin to regulate the process of transcription in its vicinity (Venkatesh and Workman, 2015). A complex combination of epigenetic modifications has been shown to regulate both ES cell identity and somatic cell reprograming by modulating chromatin conformations (Gopinathan et al., 2013; Nashun et al., 2015). For instance, it has been determined that the genome of stem cells is predominantly in the euchromatic conformation, whereas the genome of somatic cells is enriched in the heterochromatic conformation (David et al., 2011; Wang et al., 2013). In general, the distribution of trimethylation on lysine 9 and 27 of histone H3 (H3K9me3 and H3K27me3, respectively), ubiquitination on lysine 119 of histone H2A (H2AK119ub) and DNA methylation are marks related to repressed genes. In contrast, euchromatin formation is enriched in H3K4me3, H3K36me3, H3K79me3, ubiquitination of H2B, acetylation on the H3 and H4 tails, and low DNA methylation levels (Wang et al., 2013; Deng et al., 2015; Zhang T. Y. et al., 2015).

Among noncoding RNAs, micro RNAs (miRNAs) have emerged as a crucial component of the posttranscriptional regulation of gene expression in the coordination of many biological processes, including cell cycle modulation and the regulation of self-renewal, pluripotency, and differentiation (Greve et al., 2013; Mathieu and Ruohola-Baker, 2013). miRNAs are a large class of small noncoding RNAs 18–22 nt in length that bind to the 3′ UTR of their target messenger RNAs (mRNAs), leading to either translational repression or mRNA degradation (Seong et al., 2014). miRNA biogenesis starts with the transcription of the *MIR* genes, whose primary transcripts are sequentially processed by two specific endonucleases (Drosha and Dicer) to generate individual miRNAs. Once in the cytoplasm, mature miRNAs bind to Argonaute proteins to form the RNA-induced silencing complex (RISC). AGO-miRNA can recognize and suppress hundreds of mRNA targets, and one mRNA sequence can be targeted by more than one miRNA. Additionally, miRNAs are thought to be the most abundant small molecules regulating most of the genome, since more than 1,600 miRNAs have been identified in human cells (Greve et al., 2013). Therefore, miRNAs have emerged as important components of gene regulatory networks and could be involved in the establishment of particular somatic lineages (Table 3). The ways that DNA methylation/demethylation, histone modifications and miRNAs are involved in the proliferation and differentiation of human DP cells are discussed in detail below.

**Table 3:** miRNA profile of dental tissue-derived stem cells.

**Cell type**	**microRNA**	**mRNA target**	**Differentiation**	**References**
DPSCs	miR-135	Nd	Myogenic	Li et al., 2015
	miR-143			
DPSCs	miR-720	*DNMT3a, NANOG*	Osteogenic	Hara et al., 2013
PDLSCs	miR-21	*PLAP-1*	Osteogenic	Li et al., 2012
	miR-101			
DPCs	miR-424	*VEGF, KDR*	Angiogenic (endothelial cells)	Liu et al., 2014
DPSCs	miR-196	*HOX C8*	Osteogenic	Gardin et al., 2016
DPSCs	miR-218	*RUNX2*	Osteogenic	Gay et al., 2014
GMSCs				
PDLSCs				
DPSCs	miR-816-3a	*WNT5A, EGRF*	Control of cell fate	Vasanthan et al., 2015
	miR-7-5p			
DPSCs	miR-32	*DSPP*	Odontoblastic	Wang et al., 2011
	miR-586			
	miR-885-5			

*WNT5A, WNT FAMILY MEMBER 5A; EGRF, EPIDERMAL GROWTH FACTOR RECEPTOR; DSPP, DENTIN-SIALOPHOSPHOPROTEIN; DNMT3A, DNA METHYLTRANSFERASE 3A; NANOG, NANOG HOMEOBOX; PLAP-1, PERIODONTAL LIGAMENT-ASSOCIATED PROTEIN 1; VEGF, VASCULAR ENDOTHELIAL GROWTH FACTOR; KDR, VASCULAR ENDOTHELIAL GROWTH FACTOR RECEPTOR-2/KINASE INSERT DOMAIN RECEPTOR; and RUNX2, RUNT-RELATED TRANSCRIPTION FACTOR2. Nd, not determined; DFCs, dental follicle cells; DPCs, dental pulp cells; DPSCs, dental pulp stem cells; PDLSCs, periodontal ligament stem cells; GMSCs, gingival-derived mesenchymal stem cells; and SCAP, stem cells from the apical papilla*.

## Modifications of DNA in human dental tissue-derived mesenchymal stem cells

Is well-known that a subset of TFs has the ability to reprogram one type of cell into another (Takahashi and Yamanaka, 2006; Iwafuchi-Doi and Zaret, 2014). However, recent evidence indicates that cell differentiation is preceded by chromatin reorganization due to epigenetic modifications, which can either contribute to either cell stability or activate changes in cell fate (Deng et al., 2015; Rodriguez et al., 2015). On the other hand, the fact that somatic cells can be reprogramed into a pluripotent state by the ectopic expression of *OCT4, SOX2, KLF4*, and c-*MYC* suggests promising treatments for many human diseases. However, this reprogramming method is inefficient, probably due to the stochastic epigenetic events derived from DNA methylation, histone modifications, and chromatin remodeling (Takahashi and Yamanaka, 2006; Polo et al., 2012). Therefore, characterizing and understanding multiple epigenetic modifications during the establishment of cell identity and the differentiation of stem cells is an urgent research topic, and DP is considered a promising starting point. There is evidence indicating that genes required for stemness maintenance, such as *OCT4* and *NANOG*, are regulated by DNMT3a and DNMT3b. For instance, in dedifferentiated cells, the promoters of both TFs are hypomethylated, but they are re-methylated during the establishment of cell fate (Li et al., 2007). Currently, it is known that DNA methylation levels are low in pluripotent cells both *in vivo* and *in vitro*, suggesting that DNA demethylation in these cells may enhance cellular reprogramming (Polo et al., 2012; Nashun et al., 2015). For instance, third molar-derived MSCs have been reported to be more efficient at cellular reprogramming than human dermal fibroblasts, even when only three factors are introduced (*OCT3/4, SOX2*, and *KLF4*; Oda et al., 2010). By studying the CpG methylation status of the promoters of TFs *OCT3/4* and *NANOG* in parental cells, it was found that reprogramming efficiency is associated with cells with less methylation, particularly in the promoter region of *NANOG*. It was also shown that the reprogramming of DPSCs, SHED and SCAP by the forced expression of *OCT4, SOX2, KLF4*, and *LIN28* was highly efficient independently of the age or type of dental tissue. However, it was observed that the loss of DNA methylation on the *NANOG* promoter in iPSCs derived from either DPSCs or SHED was low, suggesting that *NANOG* expression is controlled by a mechanism other than DNA methylation (Yan et al., 2010). Although these studies show that dental tissues are promising sources for obtaining iPSCs, we also need to consider the suitability of their differentiation capacity and the ability of several cell-type-specific-genes to be activated and directed into different lineage cells according to the molecular signals during differentiation. In this context, although hDT-MSCs are highly proliferative and exhibit great multilineage potential, the role of DNA methylation during the establishment of cellular differentiation must be elucidated.

On the other hand, several studies have provided evidence that PDLSCs have multilineage capacity because they can develop into adipocytes and osteocytes (Table 1). However, adipogenesis can be favored over osteogenesis with the progression of aging and osteoporosis, suggesting that these conditions can lead to dysregulation of the differentiation equilibrium of MSCs (Deng et al., 2015; Avgustinova and Benitah, 2016). Another metabolic disorder that can interfere with bone remodeling is diabetes mellitus, which gives rise to high blood glucose levels (HG) and impaired bone metabolism. There is evidence that high glucose levels alter DNA methylation patterns and contribute to this disorder (Liu et al., 2016). For instance, HG treatment was found to increase *DNMT1, DNMT3a*, and *DNMT3b* expression and to induce DNA hypermethylation on PDLSCs by suppressing this form in osteoblast generation. Interestingly, the osteogenic induction from PDLSCs can be rescued by the addition of 1 μM 5-aza-2′-deoxycytidine (5-aza-dC, a DNA methyltransferase inhibitor), which decreases DNA hypermethylation levels and activates the canonical Wnt signaling pathway (Liu et al., 2016).

It was also found that application of the same concentration of 5-aza-dC in DPCs decreased their cell proliferation capacity but improved calcified nodule formation after 14 days of culture and increased the odontogenic differentiation potential of DPCs compared to that of cells cultured without 5-aza-dC (Zhang D. et al., 2015). In addition, the same authors showed that 5-aza-dC promoted the upregulation of genes encoding non-collagenous dentin matrix proteins that bind large amounts of calcium, such as *DENTIN-SIALOPHOSPHOPROTEIN* (*DSPP*) and *DENTIN MATRIX PROTEIN 1* (*DMP1*), among other TFs necessary to initiate dentin mineralization and promote increased alkaline phosphatase (ALP) activity. However, these results suggest that the DNA demethylation process, based on pharmacological assays, may provide potential strategies for dental tissue regeneration or may improve metabolic disorders, such as diabetes; thus, it is important to know which genes are affected by unbalanced DNA methylation processes and how they are affected.

Although DNA methylation is remarkably stable in human tissues, the oxidation of 5-mdC to 5-hmC is mediated by TET proteins, which act as a mechanism to regulate the balance between the gain and loss of methylation. It has been observed that hypomethylation at diverse sites of the genome, such as promoters, enhancers, TF binding sites, is correlated with the expression of genes. Therefore, TET protein functions appear to be a crucial mechanism for regulating transcription, differentiation, pluripotency, and reprogramming (Jin et al., 2014). For instance, TET1 facilitates iPSC generation by promoting the demethylation and reactivation of *OCT4* (Gao et al., 2013). Furthermore, TET1 can modulate DNA methylation levels at CpG-rich promoters participating in the repression of Polycomb target development regulators (Wu et al., 2011). Recent studies on DPCs have further revealed that TET1 may also contribute to odontogenic differentiation, as its transcript levels increase in a time-dependent manner during odontogenic development (Li et al., 2014). In addition, *TET1* knockdown expression with a short hairpin RNA decreased both mRNA and protein levels, leading to suppression of the proliferation of DPCs. In addition, *TET1* knockdown in DPCs led to low ALP activity by reducing the formation of mineralized nodules and decreasing the expression levels of *DSPP* and *DMP1*, leading to impaired odontogenic differentiation. This result suggests that TET1 may play an important role in dental repair and regeneration by modulating DNA methylation (Rao L. J. et al., 2016).

## Histone modifications involved in human dental tissue-derived cell differentiation

The amino acid residues of histones (H2A, H2B, H3, and H4) can be modified by at least 12 molecules. These modifications are reversible and are catalyzed by specific enzymes that add or remove chemical groups from the chromatin structure; their main function is to modulate TF access to the target genes (Zhang T. Y. et al., 2015). Two of the most studied histone modifications are lysine acetylation and methylation, which are essential to the regulation of many biological processes, including transcriptional regulation and cell fate decisions (Rinaldi and Benitah, 2015).

Currently, biochemical studies have revealed that histone acetyltransferases (HATs) catalyze the acetylation of the lysine (K) residues of histones H3 and H4. This modification reduces the positive charge of K, weakening its interaction with the negative charges of DNA and resulting in the unfolding of chromatin and the activation of gene transcription (Tessarz and Kouzarides, 2014). In contrast, the loss of acetylation, which is catalyzed by histone deacetylases (HDACs), leads to the heterochromatin conformation, favoring the repression of transcriptional activity (Marmorstein and Zhou, 2014). Normal HAT and HDAC activities mediate cell cycle progression, repairing DNA damage, hormone signaling and the control of cell fate, whereas the aberrant function of these enzymes impact the beginning and progression of cancer, metabolic disorders and many other diseases (Brookes and Shi, 2014).

**Figure 4:**
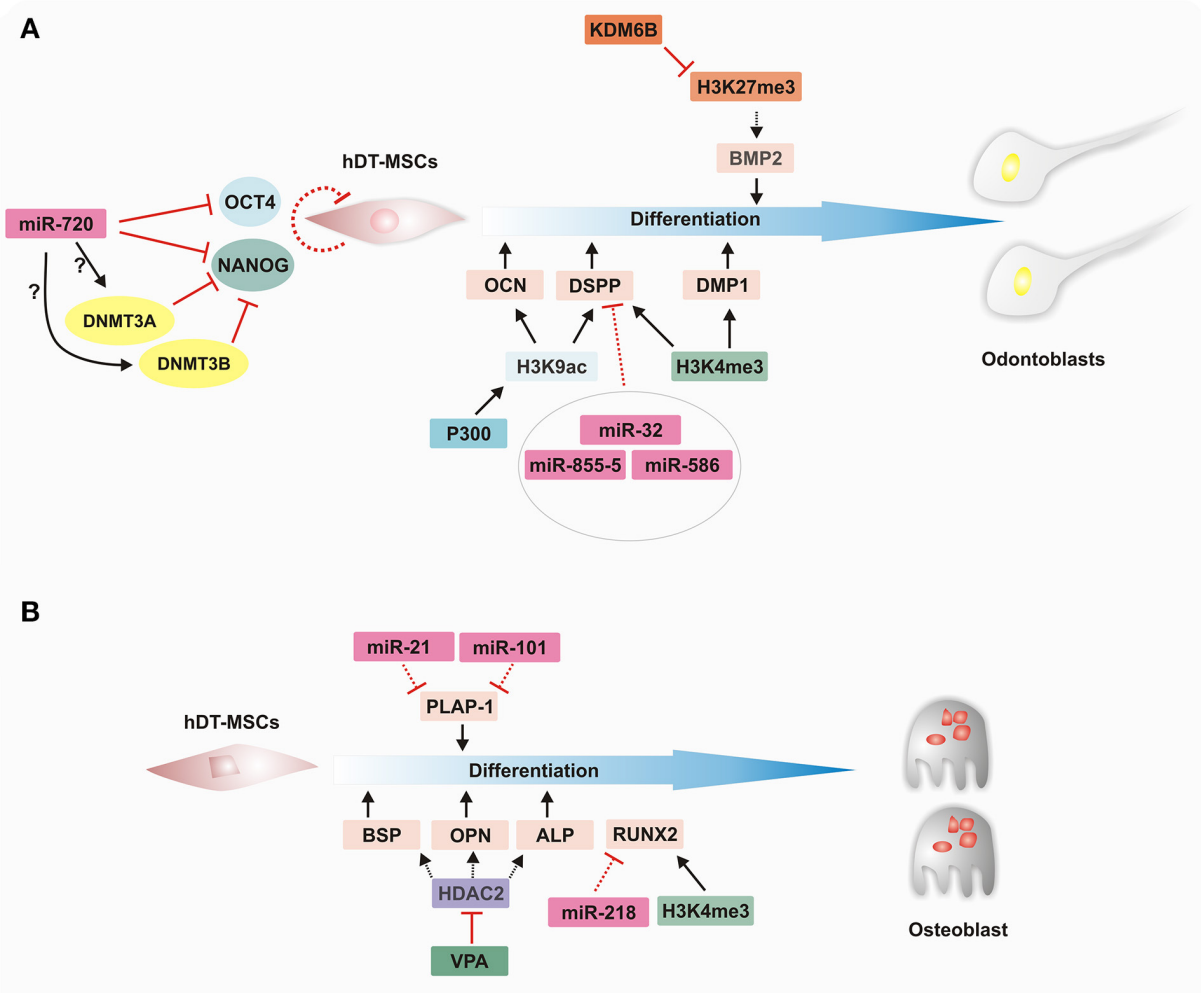
Epigenetic regulation of hDT-MSC differentiation along osteoblast and odontoblast lineages. Cell fate events are regulated by epigenetic mechanisms that modify the chromatin structure to control the transcriptional program in order to establish lineage specification. **(A)** Epigenetic landscape during odontoblast differentiation. miR720 downregulates the expression of *OCT4* and *NANOG*, whereas it induces the expression of *DNMT3a* and *DNMT3b* through an unknown mechanism to promote cell differentiation in hDT-MSCs. p300 increases the H3K9ac level in the promoter regions of *OCN* and *DSPP*, and the promoters of both *DSPP* and *DMP1* are also enriched in H3K4me3 to facilitate their expression. Furthermore, the downregulation of miRNAs levels contributes to the regulation of odontoblast differentiation by increasing *DSPP* expression, whereas *BMP2* expression levels are epigenetically controlled by KDM6B through the removal of H3K27me3. **(B)** Epigenetic regulation of osteoblastic differentiation. During induction, key transcriptional factors are activated by epigenetic regulators, such as miRNAs and histone modifiers. Decreasing levels of miR-21 and miR-101 lead to an increase in *PLAP-1* expression. On the other hand, the inhibition of HDAC by VPA enhances odontoblast differentiation by increasing *BSP, OPN*, and *ALP* expression. In addition, decreasing levels of miR-218 and the enrichment of H3K4me3 on the promoter of *RUNX2* seem to promote their expression. ALP, ALKALINE PHOSPHATASE; BMP2, BONE MORPHOGENETIC PROTEIN 2; BSP, BONE SIALOPROTEIN; DMP1, DENTIN MATRIX PROTEIN 1; DNMT3A, DNA METHYLTRANSFERASE 3A; DNMT3B, DNA METHYLTRANSFERASE 3B; DSPP, DENTIN-SIALOPHOSPHOPROTEIN; H3K4ME3, HISTONE H3 LYSINE 4 TRIMETHYLATION; H3K27ME3, HISTONE H3 LYSINE 27 TRIMETHYLATION; HDAC2, HISTONE DEACETYLASE 2; H3K9AC, HISTONE H3 LYSINE 9 ACETYLATION; KDM6B, LYSINE DEMETHYLASE 6B; miRNA, microRNA; OCN, OSTEOCALCIN; OPN, OSTEOPONTIN; PLAP-1, PERIODONTAL LIGAMENT-ASSOCIATED PROTEIN 1; RUNX2, RUNT-RELATED TRANSCRIPTION FACTOR 2; and VPA, VALPROIC ACID.

In the case of HATs, it has been shown that p300, a well-known HAT, seems to play an important role in maintaining the stemness of DPCs. The overexpression of p300 increases the transcript levels of *NANOG* and *SOX2*; however, the opposite occurs if this enzyme loses its HAT domain (Wang et al., 2014). In addition, its overexpression maintains lower expression levels of odontoblastic differentiation markers, such as *DMP1, DSPP, DSP, OPN*, and *OSTEOCALCIN* (*OCN*), suggesting that p300 has an important role and interacts with stemness markers in non-inductive conditions. Furthermore, an increase in H3K9 acetylation levels in the promoter regions of the *OCN* and *DSPP* genes has been observed when p300 is overexpressed, suggesting that p300 might act as a coactivator to regulate the odontogenic potential of DPCs (Figure 4B; Wang et al., 2014).

On the other hand, the deacetylation of histones regulates chromatin structure and transcriptional activity. However, the dysregulation of HDAC leads to diseases that impact human health. Therefore, great efforts have been focused on the generation of histone deacetylation inhibitors (HDACis), which have been used to reactivate abnormally silenced tumor suppressor genes (Marmorstein and Zhou, 2014; Gaddis et al., 2015). Other studies indicate that HDACis induce pleiotropic cellular effects and alter stem cell fate (Duncan et al., 2013, 2016b). Several types of HDACis, such as trichostatin A (TSA), valproic acid (VPA) and butyric acid, have become important in the odontogenic field, since their application promotes cell viability and reparative events in DPCs (Paino et al., 2014; Duncan et al., 2015, 2016a). In humans, there are 18 HDAC enzymes divided into four classes (Seto and Yoshida, 2014). Some members of class 1 (HDAC1, HDAC2, and HDAC3) and class 2 (HDAC4 and HDAC9) are differentially distributed in DP tissue. For instance, HDAC2 and HDAC9 are enriched in mature odontoblast nuclei but are found in distinct subnuclear structures. Even though *HDAC1, HDAC3*, and *HDAC4* show weak expression within pulp tissue, it is suggested that HDACs are involved in the transcriptional regulation of odontoblastic differentiation (Klinz et al., 2012). Jin et al. (2013) found that the application of TSA upregulated the levels of *CYCLIN D1* and *PROLIFERATING CELL NUCLEAR ANTIGEN*, leading to improved cell proliferation in DPCs. The same authors also determined that during odontoblastic induction, TSA causes *HDAC3* downregulation. This downregulation leads to increased mineralized nodule formation, promoting the expression of *DSPP, DMP1*, and *OSTEOCALCIN* (*OC*), which suggests that TSA may be used as an important accelerator of odontoblast differentiation.

Furthermore, it has also been shown that the application of VPA to DPSC cultures improves matrix mineralization during osteoblastic induction by decreasing HDAC1 and HDAC2 protein levels, which leads to an upregulation of *OSTEOPONTIN* (*OPN*) and *BONE SIALOPROTEIN* (*BSP*) mRNAs (Paino et al., 2014). By using knockdowns, the same authors determined that *HDAC2* silencing alone led to decreased levels of *OC* expression (a late stage osteoblast marker), suggesting that the specific suppression of an individual HDAC may enhance only a single aspect of osteoblast differentiation (Paino et al., 2014).

Unlike lysine acetylation, methylation does not affect the net charge of lysine. This posttranslational modification adds another level of complexity because lysine residues on histones can be monomethylated, dimethylated, or trimethylated (me1, me2, me3, respectively) by histone lysine methyltransferases. Histone methylation can affect transcription and is dependent on lysine position and methylation (Deng et al., 2015). Studies in mammalian cells have revealed that H3K4me3 enrichment is associated with the transcriptional start site of many active genes. In contrast, both H3K9me3 (mainly related to a constitutive chromatin) and H3K27me3 (a typical mark of Polycomb repressors) are marks associated with transcriptional silencing (Zhou et al., 2011). However, histone methylation is dynamically regulated by histone methyltransferases, which recognize their appropriate substrates to control transcriptional activity and other nuclear processes, such as cell cycle control and DNA repair (Van der Meulen et al., 2014; Dimitrova et al., 2015).

Currently, the functional relationship between DPC homeostasis and histone methylation is beginning to be understood. For instance, it has been determined that there are epigenetic signals that might determine the specification and differentiation of DPCs (See Table 2, Figure 4). Dangaria et al. (2011) found that dental follicle (DF) progenitor cells are characterized by high levels of H3K4me3 but that their differentiation into periodontal lineages, including alveolar bone, cementoblasts, and PDLCs, is characterized by enrichment with H3K9me3. In addition, differentiation from DF cells into osteogenic cells leads to a switch from the H3K4me3 to the H3K9me3 mark. Another more recent work supports the role of histone methylation in the cell fate of two odontogenic neural crest-derived cell types (Gopinathan et al., 2013). Using promoter-based chromatin immunoprecipitation (ChIP), the authors showed that the number of promoters enriched with H3K4me3, H3K9me3, and H3K27me3 differs slightly between DPCs and DF cells, with H3K4me3 and H3K9me3 being more prominent in DPCs and H3K27me3 particularly enriched in DF cells. Interestingly, the promoter of two odontoblast-related genes, *DSPP* and *DMP1*, was differentially regulated between DPCs and DF progenitor cells. While both promoters were enriched with H3K9me3 and H3K27me3 in DF cells, in DPCs, the same promoters were occupied by the active mark H3K4me3, which was related to the higher expression levels in DPCs than in DF cells. In addition, under mineralization induction, dynamic histone methylation was observed to be higher in DF than in DPCs. Even so, early mineralization genes, such as *RUNT-RELATED TRANSCRIPTION FACTOR2* (*RUNX2*), *MSH HOMEOBOX 2* (*MSX2*), and *DISTAL-LESS HOMEOBOX 5* (*DLX5*), were enriched with H3K4me3 in both DF cells and DPCs. On the other hand, *OSX, INTEGRIN BINDING SIALOPROTEIN* (*IBSP*), and *BONE GAMMA CABOXYGLUTAMATE PROTEIN* (*BGLAP*), a late mineralization marker, were enriched with H3K9me3 or H3K27me3. This result indicates that epigenetic regulatory mechanisms involving histone modifications are crucial in determining cell fate in odontogenic tissue-derived cells (Gopinathan et al., 2013).

Intriguingly, the removal of methyl groups from histones by histone demethylases has also been shown to contribute to the regulation of the osteogenic and odontogenic differentiation of dental MSCs. For example, Xu et al. (2013) found that histone demethylase KDM6B (also known as JMJD3), which removes H3K27me3, facilitates the expression of *BMP2* (*BONE MORPHOGENETIC PROTEIN 2*), an odontogenic master transcription gene. The same authors also showed that *KDM6B* knockdown suppressed the expression of the odontogenic marker gene *OSX* and the extracellular matrix genes *OCN* and *OPN*, significantly reducing the formation of mineralized nodules. Nonetheless, odontogenic differentiation can be rescued by the overexpression of *KDM6B* in dental MSCs with impaired *KDM6B* function. More recently, it was determined that alcohol suppresses the function of KDM6B, which leads to impaired odontogenic/osteogenic differentiation in DPSCs. The levels of several mineralization-related genes, such as *BMP2, BMP4, OCN*, and *OPN*, were decreased, probably due to the lack of erasure of H3K27me3 from its respective locus (Hoang et al., 2016). However, the overexpression of *KDM6B* showed that its function is crucial to regulating cellular differentiation, even in the presence of alcohol. Further studies must be carried out to determine whether there is a link between the beginning of osteoporosis or mineralization impairment and the dysfunction of KDM6B due to excessive alcohol consumption.

In a different study, it was found that the histone demethylase KDM2A played an important role in the regulation of *SECRETED FRIZZLED-RELATED PROTEIN 2* (*SFRP2*) during osteogenic/dentinogenic differentiation from SCAP (Yu et al., 2016). *SFRP2* regulates differentiation and tissue regeneration in dental MSCs, and its silencing induces cell death in SCAP in osteogenic-inducing medium (Fan et al., 2009). Moreover, it was found that the silencing of either *KDM2A* or *BCOR* (BCL6 co-repressor) increased the expression of *SFRP2* and the expression of osteogenic/dentinogenic markers (Fan et al., 2009). It was shown that BCOR forms a protein complex with KMD2A to regulate downstream genes, such as *EPIREGULIN* (*EREG*), and other MSC functions in SCAP (Du et al., 2013). Additionally, the loss of function of both *KDM2A* or *BCOR* leads to increased H3K36me2 and H3K4me3 levels at the *SFRP2* and *EREG* promoters, upregulating their expression. In contrast, *KDM2A* and *BCOR* overexpression, together with ChIP assays, showed that a functional protein complex can bind to the *EREG* and *SFR2* promoters to regulate their expression during osteogenic/dentinogenic differentiation in SCAP (Yu et al., 2016). Together, these results clearly support the hypothesis that histone demethylases, such as KDM6B and KDM2A, can recognize their specific substrates to properly control gene expression during cell fate transition.

## Role of miRNAs in human dental-derived mesenchymal stem cells

miRNAs drive important functions in several life processes, such as cell cycle control, and help to maintain a balance between cell proliferation and differentiation throughout development (Greve et al., 2013; Hara et al., 2013). They play an important role as regulators of gene activation or repression at specific times during the cell differentiation of dental tissue stem cells, including osteogenic, odontogenic, and angiogenic differentiation, among others. In this sense, the differential expression of miRNAs among cell populations can determine the maintenance of the stem cell phenotype or differentiation capacity, as each MSC population occupies a specific niche in dental stem cells. For instance, the miRNA profiles of DPSCs, GSCs and PDLSCs showed important changes between dedifferentiated and differentiated cell types (Gay et al., 2014). However, several miRNAs, including miR-210, miR-218, and miR-99a, may be implicated in the regulation of differentiation in the dental tissues mentioned above. It appears that miR-218 plays a key role in regulating the expression of *RUNX2*, a TF crucial for osteogenic differentiation, since there is a high correlation between lower levels of miR-218 and higher levels of *RUNX2* transcripts, suggesting that miR-218 regulates the osteogenic pathway in hDT-MSCs by modulating the expression of *RUNX2* (Gay et al., 2014). However, further studies are needed to determine how miRNA differential profiles regulate the osteogenic differentiation capacity of each hDT-MSC. Another study indicated that miR-101 and miR-21 are also involved in the osteogenic differentiation of PDLCs; both miRNAs regulate the expression of *PERIODONTAL LIGAMENT-ASSOCIATED PROTEIN 1* (*PLAP-1*) (Figure 4A) by enhancing the mineralization capacity of PDLCs (Li et al., 2012). Furthermore, DPSCs seeded on a titanium implant surface were shown to exhibit enhanced osteogenic differentiation. It seems that titanium implants induced the upregulation of miR-196a, which repressed the transcriptional activity of *HOMEOBOX C8* (*HOXC8*) and inhibited cell proliferation, promoting the osteogenic differentiation of DPSCs. This result indicates that the interaction between DPSCs and the implant surface might influence stem cell fate by upregulating miRNAs directed toward osseointegration (Gardin et al., 2016).

On the other hand, it was found that several miRNAs, including miR-32, miR-885-5, and miR-586, are differentially expressed during the odontoblast differentiation of DPCs (Figure 4B). Although these miRNAs can be found in both undifferentiated and differentiated DPCs, their levels decrease considerably under mineralization induction, which corresponds with the highest levels of *DSPP* expression, a prerequisite for initiating mineralization events (Huang et al., 2011).

It has also been found that miR-720 participates in the control of the stem cell phenotype and the differentiation of DPCs by directly repressing *NANOG* expression; it also participates indirectly by increasing the expression of *DNMT3a* and *DNMT3b* through an unknown mechanism, and these genes also act as transcriptional repressors of *NANOG*. This result is consistent with enhanced *NANOG* expression after the knockdown of miR-720, which inhibits the differentiation capacity of DPCs, whereas its overexpression induces the enhancement of *ALP* and *OPN* mRNAs by increasing odontogenic differentiation (Hara et al., 2013).

In the case of the induction of DPSCs to differentiate into the endothelial lineage, Liu et al. (2014) found that at least 29 miRNAs are altered between differentiated and undifferentiated cells in the presence of VEGF, an important pro-angiogenic factor that promotes angiogenesis. Among these miRNAs, it was found that miR-424 targeted *VEGF* and its receptor *VASCULAR ENDOTHELIAL GROWTH FACTOR RECEPTOR-2/KINASE INSERT DOMAIN RECEPTOR* (*KDR*) and regulated their translation into proteins during endothelial differentiation. Based on loss- and gain-of-function assays, the authors suggested that miR-424 might play a negative role in endothelial differentiation because its inhibition promotes the transcript and protein accumulation of both VEGF and KDR, which might contribute to DP repair and regeneration.

The function of miRNAs during the myogenic differentiation of DPSCs has also been reported (Li et al., 2015). It was found that the application of 5-aza-dC impaired the function of both miR-135 and miR-143 and arrested cell proliferation to sustain myogenic differentiation, indicating that miRNAs could perform a decisive function in DPSC fate. miR-516a-3p and miR-7-5p have also been identified as important regulators of cell fate decisions and self-renewal, respectively, by targeting *WNT FAMILY MEMBER 5A* (*WNT5A*) and *EPIDERMAL GROWTH FACTOR RECEPTOR* (*EGFR*) mRNAs. Therefore, the presence of both miRNAs in DPSCs may influence the transition from an undifferentiated state to a differentiated state or may maintain the cells in a quiescent state (Vasanthan et al., 2015). All these data show that miRNAs are required to shape dental MSCs into several somatic lineages. Therefore, they can be used to increase the efficiency and quality of lineages in future therapeutic applications.

## Concluding remarks

Dental tissue-derived mesenchymal stem cells are currently a topic of extensive research. They offer an excellent source of stem cells that can be easily obtained in a minimally invasive way without ethical concerns. Dental stem cells exhibit self-renewal and a high potential to differentiate into multiple cell lineages (Figure 2). The ability to generate diverse cell types could provide hope for the treatment of several disorders and diseases. The integration and function of hDT-MSC derivatives have been tested in animal models and appear to be encouraging for liver and vision disturbances, neurological disorders, heart failure, muscular dystrophy, and other diseases. Unfortunately, the use of hDT-MSCs in clinical trials to treat human diseases, such as periodontitis or dental pulp diseases, has failed (Yang et al., 2017). This application is a field of opportunity, but additional work is required to understand the biology and true regenerative potential of MSCs derived from the oral cavity. Efforts might be focused on generating suitable cell types for specific disorders or diseases.

In recent years, epigenetic mechanisms have been a growing topic in the pursuit of understanding mesenchymal stem cell function and cell plasticity. In this sense, the epigenetic mechanisms of DNA, histones, and miRNAs as well their respective modifiers are critical to specifying accurate gene expression patterns and cellular function. However, environmental stressors and aging (among other events) can dysregulate the function of epigenetic factors, affect the function of stem cells and even lead to the initiation of several diseases. For example, increased expression levels of *DNMT1* or *DNMT3A*, which are caused by high glucose levels in the blood, impair osteogenic differentiation, whereas the overexpression of *EHZ2*, a component of PRC2, or the loss of function of KDM6B favors adipogenic differentiation over osteogenesis (Deng et al., 2015; Liu et al., 2016). Therefore, a deeper understanding of chromatin regulation during the establishment of cell fate specification could provide a good option for the treatment of bone-related defects and other metabolic disorders.

However, for the regeneration of disease-specific cell types, additional work is still required to understand the differentiation programs and true regenerative potential of dental stem cells. First, we must consider that every tissue-specific stem cell contains unique epigenetic modifiers, whereas other modifiers are ubiquitously expressed. Additionally, both specific and ubiquitous modifiers can target many additional genes. Based on these statements, the following question remains: what are the similarities and particularities of epigenetic remodeling during lineage specification among different dental stem cells? Moreover, although epigenetic marks deposited by chromatin-modifier enzymes have been correlated with cellular phenotype, what truly decisive contributions to individual epigenetic marks or their combinations make during the transition of MSCs to cellular differentiation? Additionally, several epigenetic modifiers can recognize the same epigenetic marks. However, whether these modifiers exert an orderly temporal function or perform independent fine modulation cell fate still remains largely unexplored. In conclusion, an understanding of epigenetic modifications in the transcription control, maintenance, and cellular differentiation of hDT-MSCs could serve as a starting point for regenerative medicine.

## Author contributions

BR-J and MC-C: summarized the literature and wrote the manuscript; RR-H and CD-l-P critically revised and wrote part of the manuscript; GN-C: conceptualized and planned the manuscript and prepared the figures; All authors agree to be accountable for the content of this work.

### Conflict of interest statement

The authors declare that the research was conducted in the absence of any commercial or financial relationships that could be construed as a potential conflict of interest. The reviewer MLN and handling Editor declared their shared affiliation.
